# Prophylactic Antibiotics before Gynecologic Surgery: A Comprehensive Review of Guidelines

**DOI:** 10.3390/jpm14030327

**Published:** 2024-03-21

**Authors:** Stamatios Petousis, Panagiota Angelou, Aristarchos Almperis, Antonio Simone Laganà, Gerasimos Titilas, Chrysoula Margioula-Siarkou, Konstantinos Dinas

**Affiliations:** 1 Gynaecologic Oncology Unit, 2nd Department of Obstetrics and Gynaecology, Aristotle University of Thessaloniki, 541 24 Thessaloniki, Greece; 2 Unit of Obstetrics and Gynecology, “Paolo Giaccone” Hospital, Department of Health Promotion, Mother and Child Care, Internal Medicine and Medical Specialties (PROMISE), University of Palermo, 90127 Palermo, Italy; antoniosimone.lagana@unipa.it

**Keywords:** antibiotics, gynecologic surgery, guidelines, prophylaxis

## Abstract

Surgical site infections (SSIs) refer to infections in the incision, organ, or postoperative space. As common healthcare-associated infections, SSIs correlate with prolonged hospital stay, additional procedures, ICU stay, and higher mortality rates. Around 8–10% of gynecologic surgery patients may experience infectious complications, influenced by microbial contamination, surgical nature, and patient factors. The goal of this narrative review is to compare and merge recommendations from globally published guidelines concerning the utilization of antibiotics in the perioperative phase. A comparative descriptive/narrative review of the guidelines issued by the American College of Obstetrics and Gynecology (ACOG), Society of Obstetricians and Gynecologists of Canada (SOGC), Royal College of Obstetricians and Gynecologists (RCOG), National Institute for Health and Care Excellence (NICE), Royal Australian and New Zealand College of Obstetricians and Gynecologists (RANZCOG), European Society of Gynecologic Oncology (ESGO), Société Française d’ Anésthesie et de Réanimation (SFAR), Spanish Society of Infectious Diseases and Clinical Microbiology (SEIMC), and Hellenic Society of Obstetrics and Gynecology (HSOG) was conducted. For hysterectomy, first/second-generation cephalosporins are suggested, with metronidazole as an option. Laparoscopy without entering the bowel or vagina typically does not require prophylaxis. Uterine evacuation and hysteroscopy may involve doxycycline or azithromycin based on risk factors, whereas, for vulvectomy, cefazolin is recommended. Urogynecology procedures may include cefazolin with metronidazole. In cases of penicillin allergy, cephalosporins are suggested, and, for obese patients, adjusted doses are advised. Additional doses may be needed for prolonged procedures or excessive blood loss. Timing recommendations are 15–60 min before incision, adjusting for specific antibiotics. Clear indications exist for certain surgeries like hysterectomy, termination of pregnancy, and urogynecologic procedures. Conversely, procedures such as intrauterine device insertion, hysteroscopy, and laparoscopy typically do not necessitate antibiotic prophylaxis. For several other procedures, the evidence is inconclusive, while considering dose, timing, and indications can mitigate infectious complications and provide benefits for the healthcare system.

## 1. Introduction

Surgical site infections (SSIs) are defined as infections that occur after surgery and concern either the incision or the internal organs and structures close to the part of the body where the surgery took place [1]. They are some of the most frequent healthcare-associated infections (HAIs) and are associated with longer postoperative hospital admissions, additional surgical operations, treatment in critical care units, and increased death rates [2]. It is estimated that 8–10% of patients undergoing gynecologic surgery will develop infectious complications, such as infection of the urinary tract, the surgical wound, or the perineum, endometritis, vaginal cuff cellulitis, and sepsis [3]. The risk of developing a postoperative infection depends on various factors, including the inherent level of microbiological contamination of the surgical site, the nature of the surgical procedure, and specific patient variables and clinical features.

The majority of SSIs in gynecologic surgery are caused by pathogens from the patient’s skin or vaginal flora. These organisms are mainly aerobic Gram-positive cocci (e.g., staphylococci), but, when the incision is made close to the perineum or groin, they may contain fecal flora (e.g., anaerobic bacteria and Gram-negative aerobes). Moreover, in surgical procedures in which the vagina is open, the surgical field is in danger of contamination with many aerobic or anaerobic microorganisms from its indigenous flora. In order to classify the condition of wounds based on the degree of intraoperative microbial contamination, the CDC has established classification definitions composed of four classes, which include the following: clean, clean–contaminated, contaminated, and dirty–infected [Table 1] [4]. In gynecology, laparotomies and laparoscopies in which the vagina is not breached are classified as clean procedures, whereas procedures such as hysterosalpingography (HSG), sonohysterography, intrauterine device (IUD) insertion, and endometrial biopsy, which breach the endocervix, are considered as clean–contaminated procedures.

Additional factors related to the operative procedure, such as the length of the surgical scrub, skin antisepsis, preoperative shaving, procedure duration, ventilation in the operating room, insufficient sterilization of instruments, use of foreign materials, surgical drains, and surgical technique, may also increase the risk of surgical site infections (SSIs) [5]. In addition, when assessing the risk of an infectious complication after an operation, the integrity of the patient’s immune system should be taken into consideration. Chronic illnesses, malnutrition, hyperglycemia, obesity, smoking, prolonged intake of immunosuppressive drugs, as well as the presence of an additional infection are all conditions that impair the host’s defenses and, therefore, are related to increased rates of SSIs [6].

Surgical antibiotic prophylaxis is defined as the use of antibiotics in order to forestall the appearance of an infection at the surgical site [7]. The goal of administering antibiotic prophylaxis is to lower the colonization pressure of the microorganisms that are introduced during the procedure to a level where the patient’s immune system is capable of combating potential illnesses [4]. It should be highlighted that prophylactic antibiotics neither sterilize the tissues nor treat a preexisting infection. The agents that are eligible for use must be scientifically proven to reduce the rate of postoperative infections, safe, and cost-effective. It is also necessary that they achieve sufficient serum and tissue levels prior to making the incision and maintain a curative effect for a short period of time after the end of the surgical procedure [4].

In many countries across the world, various institutions have published different guidelines concerning the optimal preoperative care for gynecologic surgical procedures based on the most up-to-date available data. Taking into consideration all of the above, an effort to compile and succinctly summarize all the current guidelines concerning the use of prophylactic antibiotics prior to gynecologic surgical procedures could be highly beneficial and further facilitate the establishment of broadly accepted evidence-based clinical practice principles. The purpose of this narrative review is to evaluate, compare, and synthesize recommendations from published international guidelines concerning the use of antibiotics in the preoperative period.

## 2. Materials and Methods

The aim of the present descriptive review was to evaluate existing guidelines published by internationally acknowledged medical organizations, colleges, associations, societies, committees, and study groups regarding the use of prophylactic antibiotics for gynecologic surgeries. This review was conducted following the preferred reporting items for systematic reviews and meta-analysis (PRISMA) recommendations. A search of the literature was conducted in August 2023 through PubMed and Scopus databases. The literature search included the period 1990–2023. The electronic search was conducted by using a combination of terms “antibiotics”, “gynecologic surgery”, and “guidelines” combined with “AND” operator. The search was conducted for terms included in the title, abstract, or keywords. Additionally, the websites of internationally renowned medical organizations and societies with scientific interest regarding gynecology, surgery, and infectious diseases were also searched in order to identify official published guidelines pertinent to the goal of the present review. Namely, American College of Obstetrics and Gynecology (ACOG), Society of Obstetricians and Gynecologists of Canada (SOGC), Royal College of Obstetricians and Gynecologists (RCOG), National Institute for Health and Care Excellence (NICE), Royal Australian and New Zealand College of Obstetricians and Gynecologists (RANZCOG), European Society of Gynecologic Oncology (ESGO), Société Française d’ Anésthesie et de Réanimation (SFAR), Spanish Society of Infectious Diseases and Clinical Microbiology (SEIMC), and Hellenic Society of Obstetrics and Gynecology (HSOG) were reviewed.

The reference lists of the included studies were also searched manually to retrieve additional relevant articles. Exclusion criteria included all other types of studies, except for official guidelines, as well as guidelines written in any language other than English, French, and Greek. The choice of languages was based on the linguistic fluency of the authors.

**Figure 1:**
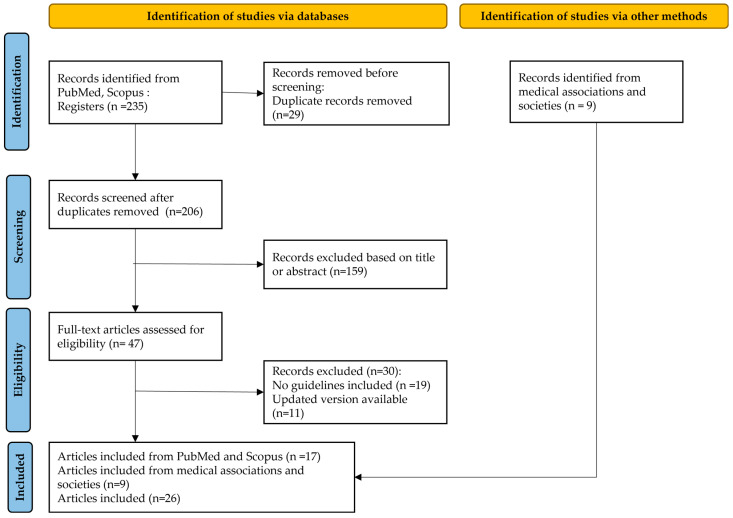
Flowchart of study selection.

Systematic search revealed 235 items with potential for inclusion in our narrative review. At first, two investigators (S.P. and P.A.) independently screened records for duplicates and eligibility based only on titles and abstracts (28 were duplicated and another 159 were excluded based on title or abstract). Consequently, full texts of the potentially eligible articles were screened independently by each investigator for final inclusion (47 items assessed as potentially eligible for our review). Furthermore, 19 items were excluded because of not reporting guidelines and 11 items were excluded because of updated versions available. Moreover, 9 items were identified and included from the websites of internationally recognized medical organizations and societies. There were finally 26 published guidelines regarding the use of prophylactic antibiotics in gynecologic surgical procedures that were retrieved and included in the present review. The flowchart of study selection is presented in Figure 1.

## 3. Results

### 3.1. Antibiotic Prophylaxis According to the Type of Gynecologic Procedure

#### 3.1.1. Vaginal, Abdominal, or Laparoscopic Hysterectomy

Patients undergoing vaginal, abdominal, or laparoscopic hysterectomy should receive a single dose of antimicrobial prophylaxis. The antibiotic of choice varies among different guidelines. The use of first- or second-generation cephalosporins (such as cefazolin, cefamandole, and cefuroxime) is recommended by the ACOG, SOGC, SFAR, and Surgical Infection Prevention Guideline Writers Workgroup (SIPGWW) [8,9,10,11]. The RANCOG and the Surgical Antibiotic Prophylaxis Guideline at the Women’s in Parkville and in Sandringham suggest the addition of metronidazole to cefazolin as antibiotic prophylaxis prior to hysterectomy [12,13]. Moreover, the use of cefazolin can be replaced by an aminopenicillin with a β lactamase inhibitor, such as amoxicillin–clavulanic acid, as stated by the SEIMC and the Enhanced Recovery After Surgery (ERAS) protocol [14,15]. Finally, according to other guidelines, antibiotic prophylaxis should be differentiated depending on the type of hysterectomy. To be more precise, the HSOG suggests the use of a first/second-generation cephalosporin or amoxicillin–clavulanic acid for abdominal hysterectomy and a first-generation cephalosporin (cefazolin) for laparoscopic and vaginal hysterectomy [16], while earlier published guidelines supported the use of a first-generation cephalosporin (cefazolin, cefotetan, or cefoxitin) for abdominal and the use of a cephalosporin with anaerobic activity (cefotaxime or cefazolin) or amikacin for vaginal hysterectomy [17]. The International Society for Gynecologic Endoscopy (ISGE) proposes the use of cephalosporins (second or third generation) and metronidazole for vaginal hysterectomy [18].

#### 3.1.2. Laparotomy without Entry into Bowel or Vagina

Laparotomy without entry into the bowel or vagina is considered to be a clean procedure. The National Institute for Health and Care Excellence does not recommend antibiotic prophylaxis for clean surgeries, unless they involve placement of a prosthesis or implant [19]. However, according to the ACOG, a single dose of antibiotic prophylaxis with 2 g of cefazolin intravenously may be considered based on scant data that indicate a benefit [8]. In addition, the RANCOG and Surgical Antibiotic Prophylaxis Guideline at the Women’s in Parkville and in Sandringham suggest the use of cefazolin (dose 2 g intravenously) combined with metronidazole (dose 500 mg intravenously) for patients undergoing major abdominal procedures [12,13].

#### 3.1.3. Laparoscopy without Entry into Bowel or Vagina

Laparoscopy without entry into the bowel or vagina is considered to be a clean procedure. There is a consensus among all the guidelines that antibiotic prophylaxis is not recommended [8,9,12,13,14,15,16,17].

#### 3.1.4. Diagnostic or Operative Hysteroscopy

Antibiotic prophylaxis is not recommended for routine hysteroscopic procedures in women with no risk factors [8,9,10,12,13,16,20]. The Surgical Antibiotic Prophylaxis Guideline at the Women’s in Parkville and in Sandringham points out the need to administer azithromycin for patients with dilated tubes or a history of pelvic inflammatory disease.

#### 3.1.5. Hysterosalpingography

Routine use of antibiotic prophylaxis is not recommended for patients undergoing hysterosalpingography without any risk factors [8,9,10,12]. On the other hand, antibiotic therapy should be instituted if there is a history of pelvic inflammatory disease or the fallopian tubes are noted to be abnormal at the time of the procedure (e.g., dilated). In these cases, doxycycline [8,9,16] or azithromycin (dose 1 g orally) [13] should be prescribed. 

#### 3.1.6. Intrauterine Device Insertion

Antibiotic prophylaxis is not recommended for routine insertion of an intrauterine device. However, according to the RCOG, the Surgical Antibiotic Prophylaxis Guideline at the Women’s in Parkville and in Sandringham, and the HSOG, healthcare professionals could consider screening for sexually transmitted infections, such as *C. trachomatis*, *N. gonorrhoeae*, *M. genitalium*, and bacterial vaginosis, in high-risk populations and administer the appropriate treatment [9,13,16].

#### 3.1.7. Cervical Tissue Excision Procedures (e.g., Large Loop Excision of Transformation Zone)

The RANCOG and the ACOG conclude that there is no evidence of a reduction in infection rates with antibiotic prophylaxis for patients undergoing cervical excision procedures, including large loop excision of transformation zone (LLETZ). Therefore, antibiotic prophylaxis is not recommended unless there is a reason to suspect higher infection risk [8,12].

#### 3.1.8. Uterine Evacuation

The ACOG suggests the use of prophylactic antibiotics for patients undergoing uterine evacuation for induced abortion, early pregnancy loss, and for second trimester dilation and evacuation. The antibiotic of choice is doxycycline (a single dose of 200 mg is recommended 1 h before uterine aspiration). Metronidazole is an acceptable second-line agent [8]. The use of prophylaxis is also supported by the RANCOG, the SOGC, and the HSOG for patients who have not been adequately investigated before surgical termination of pregnancy. The antibiotic of choice is doxycycline; however, the suggested dose is 100 mg orally prior to the procedure and 200 mg orally after the procedure [9,12,16]. Alternative regimens include the administration of 400 mg of doxycycline orally with food 10–12 h prior to the procedure [12,16], or 2 g of metronidazole orally combined with 1 g of azithromycin orally prior to the procedure for patients at high risk of infection [12], or 400 mg of metronidazole before and repeat dosage 4 and 8 h after the procedure [16]. The SEIMC and AEC, for patients undergoing induced abortion or puerperal curettage, recommend the use of doxycycline 100 mg orally or intravenously prior to the procedure. An alternative option includes the use of azithromycin 1 g orally or intravenously combined with metronidazole 500 mg orally [14]. On the other hand, the SOGC does not support the use of prophylactic antibiotics to lower infectious morbidity after surgery for a missed or incomplete abortion [9], while the SFAR does not support the use of prophylaxis for surgical termination of pregnancy [10]. 

#### 3.1.9. Endometrial Biopsy

Among the guidelines, there is unanimous agreement that the available evidence advocating for antimicrobial prophylaxis prior to endometrial biopsy is deemed inadequate. Consequently, it does not attain the status of standard care [8,9,10,13,16]. 

#### 3.1.10. Vulvectomy

According to the Enhanced Recovery After Surgery Society (ERAS) guidelines for optimal perioperative care for vulvar and vaginal surgeries, there are no randomized trials supporting antimicrobial prophylaxis in patients undergoing a vulvectomy. However, considering the high incidence of SSI, it seems reasonable to administer a single dose of antibiotic [21]. The ACOG classifies this procedure as clean–contaminated and, in this context, recommends 2 g of cefazoline intravenously [8]. 

#### 3.1.11. Urogynecology Procedures

For urogynecological procedures, the RANCOG, the HSOG, and the Surgical Antibiotic Prophylaxis Guideline at the Women’s in Parkville and in Sandringham recommend the use of 2 g of cefazolin combined with 500 mg of metronidazole [12,13,16].

Patients undergoing transvaginally placed slings are candidates for antimicrobial prophylaxis [8,9,10]. More particularly, the ACOG and the SOGC suggest 2 g of cefazolin intravenously, while the SFAR calls for the use of 2 g of aminopenicillin with a β lactamase inhibitor intravenously. On the contrary, the ERAS protocols conclude that there is insufficient evidence to make concrete recommendations. Taking that into consideration, 93% of the surgeons surveyed reported using antibiotic prophylaxis [21].

According to the ACOG, patients undergoing anterior or posterior colporrhaphy could benefit from antimicrobial prophylaxis because the surgical wound that results from the incision of the vaginal epithelium is categorized as clean–contaminated [8]. However, the ERAS cannot present enough evidence to make specific recommendations [21].

As for women with no additional risk factors undergoing cystoscopy, antibiotic prophylaxis is not recommended by the guidelines, provided they have negative urine cultures [8,10,14].

#### 3.1.12. Urodynamic Studies

The existing guidelines do not recommend antibiotic prophylaxis for urodynamic studies [8,9,10,14,16].

An overview of all the recommendations included for each surgical procedure is presented in Table 2.

### 3.2. Special Cases in Antibiotic Prophylaxis

#### 3.2.1. Hypersensitivity to Penicillin

The rate of patient-reported penicillin allergy ranges from 8 to 15% in the United States. However, only 5% of these patients have a true IgE-mediated allergy, including anaphylaxis, which is the most life-threatening immediate presentation of an allergic reaction, when tested [22]. Adverse effects of penicillin are considered to be associated with the formation of antibodies against metabolites of the penicillin molecule, specifically the R1 side chains of the β lactam ring [15]. Cefazolin, the most commonly used antibiotic agent for prophylaxis in gynecologic surgery, does not share the R1 chain with penicillin, and, based on a systematic review and meta-analysis, less than 1% of patients with an unconfirmed penicillin allergy and 3% of patients with a confirmed history of allergic reaction experience hypersensitivity reactions to cefazolin [23]. In this context, the standard antibiotic prophylaxis regimen, which may include cefazolin or ertapenem if needed, is advised for patients with a documented penicillin allergy, according to the Enhanced Recovery after Surgery (ERAS) Society guidelines for gynecologic oncology. The ACOG, RANCOG, and SOGC also recommend cephalosporin prophylaxis for patients with a confirmed history of immediate or delayed non-severe allergy to penicillin [8,9,12]. On the other hand, alternative antibiotics should be considered for patients that have experienced severe hypersensitivity to penicillin, such as anaphylaxis or exfoliative dermatitis (Stevens–Johnson syndrome and toxic epidermal necrolysis). Clindamycin, when administered intravenously, has been proven to be safe and effective and can be used either alone (dose 600 mg [9] or 900 mg [8]) or in combination with gentamicin (dose 2 mg/kg) [11,12,13,14], erythromycin (dose 500 mg) [16], aztreonam, or ciprofloxacin [11]. Another widely acceptable antibiotic is metronidazole (dose 500 mg), which can be used as a monotherapy [9] or in combination with gentamycin (dose 5 mg/kg) [8,11] or ciprofloxacin [11]. Additionally, alternative regimens include aztreonam [8], erythromycin [9], and vancomycin with gentamycin [14]. Finally, the SFAR provides recommendations based on the type of surgical procedure, suggesting the administration of clindamycin with gentamicin for hysterectomy and gentamicin with metronidazole for prolapse surgery [10].

#### 3.2.2. Obese Patients

In obese patients, the higher rate of surgical-associated infections, as well as the pharmacokinetic alterations that can modify the tissue concentration of some antibiotics, have raised the question of increasing or repeating the dosage of antimicrobial prophylaxis in this patient category. In this context, the ACOG and the RANCOG recommend a 3 g intravenous dose of cefazolin for patients weighing more than 120 kg [8,12]. In addition, the SOGC, the SFAR, and the HSOG recommend doubling the usual dose of prophylaxis for patients with morbid obesity (BMI > 35 kg/m^2^) based on expert opinion [9,10,16]. Similarly, the ERAS suggests that the dose be increased in obese patients (BMI > 35 kg/m^2^ or >100 kg) [15], while the SEIMC and AEC point out the need to use adjusted and ideal body weight, rather than actual body weight, in order to more accurately correct the dose in obese patients [14]. Finally, even though surgeons can consider using published weight-based dosing of the antibiotic needed, the data to support redosing antibiotics intraoperatively or continuing postoperatively based solely on obesity are insufficient [24].

#### 3.2.3. Prolonged Procedures

For prolonged procedures, additional intraoperative doses of an antibiotic should be administered in order to maintain adequate levels throughout the operation. The ACOG, SEIMC and AEC, ERAS, NICE, SIGO, Consensus Bundle on Prevention of Surgical Site Infections After Major Gynecologic Surgery, and the Surgical Antibiotic Prophylaxis Guideline at the Women’s in Parkville and in Sandringham agree on administering an additional dose of antibiotic prophylaxis when the operation is longer than the half-life of the antibiotic administered [8,13,14,15,19,25]. Similarly, according to the RANCOG, the SOGC, and the HSOG, a repeat dose should be administered if the procedure lasts over 3 h [9,12,16].

#### 3.2.4. Excessive Blood Loss

There is a consensus between the existing guidelines that a second intraoperative dose of the antibiotic agent should be administered when the estimated blood loss is higher than 1500 mL [8,9,10,14,15,16,25,26].

#### 3.2.5. Colonization or High Risk of Colonization with MRSA

It is advised that patients with a history of Methicillin-Resistant Staphylococcus Aureus (MRSA) colonization or infection who are undergoing a surgical procedure follow a hospital-based MRSA antibiotic prophylaxis protocol or adjust the standard prophylactic antibiotic regimen by adding a preoperative dose of 15 mg/kg of vancomycin intravenously [8,12,13]. The SEIMC and AEC also suggest that the use of glycopeptides be considered in patients with a history of colonization or infection by MRSA or in cases of hospitals with a high MRSA prevalence rate [14]. Finally, according to the Surgical Infection Prevention Guideline Writers Workgroup (SIPGWW), while, for patients with a confirmed MRSA colonization, vancomycin could be utilized as the antimicrobial agent of choice for prophylaxis, there is insufficient evidence to support its use for patients at high risk of colonization with MRSA [11].

A summary of all the above recommendations is presented in Table 3.

### 3.3. Time of Administration of Antibiotic Prophylaxis

The goal of administering prophylactic antibiotics before a surgical procedure is to prevent the appearance of an infection at the surgical site. In order to achieve that, adequate serum and tissue levels should be reached before the incision is made, and the curative effect must be maintained for a short period of time after the end of the surgical procedure. Therefore, if antibiotic prophylaxis is advisable, it should be administered promptly before or during bacterial inoculation [9]. This should be conducted 15 to 60 min [8,11,13,14,15,16,17,25,26], ideally at least 30 min [10,12], before skin incision, usually at the time of anesthesia induction. Moreover, the guidelines suggest taking into consideration the timing and pharmacokinetics (such as the serum half-life), as well as the necessary infusion time of the antibiotic [8,10,11,13,14,18,25,26,27]. Accordingly, if quinolones, aminoglycosides, or vancomycin are recommended, a timeframe of up to 2 h before surgical incision is allowable [8,10,11,14,22,25]. An overview regarding time of administration of antibiotic prophylaxis is presented in Table 4.

## 4. Discussion

The strengths of this review include the synthesis of the major guidelines in order to offer clinicians easily accessible and evidence-based information that they can use in their routine practice so as to make decisions rooted in scientific research, standardize medical care, and improve patient outcomes. However, guidelines can vary across different organizations and regions, making it challenging to arrive at a universal conclusion. After conducting a comprehensive review of all the guidelines included in this article regarding the prophylactic use of antibiotics in gynecologic surgery, we come to the conclusion that, while for certain surgical procedures (e.g., hysterectomy, hysteroscopy, and laparotomy without entry into the bowel or vagina) there are no notable variations among the different companies, for others (e.g., uterine evacuation and urogynecology procedures), there are some inconsistencies or conflictions in recommendations from different sources. It is worth mentioning that some affiliations did not provide specific guidelines for each type of gynecologic procedure but only analyzed the basic principles and general recommendations concerning antibiotic prophylaxis regimens. The lack of available data creates a need for further research in order to obtain enough high-quality evidence-based information. In addition, the items included in this review were retrieved from a literature search conducted through PubMed and Scopus, and some of the websites of internationally recognized medical organizations and societies with scientific interest in gynecology, oncology, surgery, and infectious diseases that provided guidelines in English, French, or Greek. Therefore, more national associations and companies may have issued relevant guidelines that were not included in this review. It is important to note that all the included guidelines concern developed countries, where the SSI rates are significantly lower compared to those recorded in developing countries. The studies conducted in African countries demonstrated increased incidence of SSIs [28], and the highest rate, according to the latest data, was reported in Ethiopia, where the prevalence of SSIs is up to 25.2% and adherence to surgical antibiotic prophylaxis guidelines is poor [29,30]. In 2018, Ethiopia’s Ministry of Health (MOH) proposed a practical guide on hospital administration of antibiotics, which was implemented in many institutions by 2022. These guidelines were in accordance with the ones issued by other international organizations mentioned in this review [30,31]. However, the progress was not as expected due to medication shortages, insufficient cooperation between healthcare professionals, lack of funding, poor ownership, and high rates of staff turnover. Therefore, we can come to the conclusion that creating guidelines based exclusively on scientific knowledge and evidence-based medicine for the ideal regimen of antimicrobial prophylaxis prior to gynecologic procedures is inadequate. It is crucial to address other elements and make sure that these recommendations are in fact applicable in clinical practice. More specifically, the availability of the medications proposed, cost-effectiveness, compliance with other intraoperative measures for prevention of infections, and the national- or hospital-based bacterial resistance patterns should be taken into account.

## 5. Conclusions

The goal of this narrative review was to summarize all the existing guidelines regarding the prophylactic use of antibiotics in gynecologic surgery. Antibiotic prophylaxis has a significant impact on outcomes in procedures with high postoperative infection rates. For some procedures, such as hysterectomy, surgical termination of pregnancy, and urogynecologic surgery, antibiotic prophylaxis is undoubtedly recommended. For other procedures, such as insertion of an intrauterine device, hysteroscopy, and laparoscopy (without entry into bowel or vagina), antibiotic prophylaxis is typically not required. For several other procedures, the evidence for prophylactic antibiotics is either insufficient or unclear. Administering the appropriate antibiotic, at the correct dose and timing, with the adequate frequency and for the right indications, will reduce patient morbidity, caused by infectious complications, and increase benefits for the healthcare system.

## Figures and Tables

**Table 1:** Surgical Wound Classification Reproduced from Mangram AJ, Horan TC, Pearson ML, et al. Guideline for Prevention of Surgical Site Infection, 1999. Centers for Disease Control and Prevention (CDC) Hospital Infection Control Practices Advisory Committee. Am. J. Infect. Control 1999, 27, 97–132 [4].

Class 1 wounds (Clean)	An uninfected operative wound in which no inflammation is encountered and the respiratory, alimentary, genital, or uninfected urinary tract is not entered. In addition, clean wounds are primarily closed and, if necessary, drained with closed drainage. Operative incisional wounds that follow nonpenetrating (blunt) trauma should be included in this category if they meet the criteria.
Class 2 wounds (Clean–contaminated)	An operative wound in which the respiratory, alimentary, genital, or urinary tracts are entered under controlled conditions and without unusual contamination. Specifically, operations involving the biliary tract, appendix, vagina, and oropharynx are included in this category, provided no evidence of infection or major break in technique is encountered.
Class 3 wounds (Contaminated)	Open, fresh, accidental wounds. In addition, operations with major breaks in sterile technique (e.g., open cardiac massage) or gross spillage from the gastrointestinal tract, and incisions in which acute nonpurulent inflammation is encountered are included in this category.
Class 4 wounds (Dirty–infected)	Old traumatic wounds with retained devitalized tissue and those that involve existing clinical infection or perforated viscera. This definition suggests that the organisms causing postoperative infection were present in the operative field before the operation.

**Table 2:** Overview of all the recommendations included for each surgical procedure.

Issued	The Royal Australian and New Zealand College of Obstetricians and Gynecologists (RANCOG) 2021	American College of Obstetricians and Gynecologists (ACOG) 2018	Society of Obstetricians and Gynecologists of Canada (SOGC) 2018		Société Française d’ Anésthesie et de Réanimation (SFAR) 2017
Hysterectomy: Laparotomy Laparoscopy Vaginal	Cefazolin 2 g iv PLUS metronidazole 500 mg iv	Cefazolin 2 g iv	Single dose of first- or second-generation cephalosporin iv		Cefazolin 2 g iv Cefamandole 1.5 g iv Cefuroxime 1.5 g iv
Laparotomy without entry into bowel or vagina	Cefazolin 2 g iv PLUS metronidazole 500 mg iv	Consider cefazolin 2 g iv	NR		NR
Laparoscopy without entry into bowel or vagina	Not recommended	Not recommended	Not recommended		Not recommended
Hysteroscopy: Diagnostic Operative	Not recommended	Not recommended	Not recommended		Not recommended
Hysterosalpingography	Not recommended ^1^	Not recommended ^1^	Insufficient evidence ^3^		Not recommended
Intrauterine device insertion	Not recommended	Not recommended	Not recommended ^4^		Not recommended
Endometrial biopsy	NR	Not recommended	Insufficient evidence		Not recommended
Cervical tissue excision procedures (e.g., Large loop excision of transformation zone)	Not recommended	Not recommended	NR		NR
Uterine evacuation	Surgical termination of pregnancy when STIs have not been excluded: doxycycline 100 mg po before and 200 mg po after the procedure OR doxycycline 400 mg po with food 10–12 h prior to the procedure ^2^	Suction D&C, D&E: Doxycycline 200 mg D&C for nonpregnancy indications: Not recommended	Induced (therapeutic) surgical abortion: Doxycycline 100 mg po pre-procedure PLUS 200 mg po post-procedure Surgery for a missed or incomplete abortion: Not recommended		Surgical termination of pregnancy: Not recommended
Vulvectomy	NR	Cefazolin 2 g iv	NR		NR
Urogynecology procedures	cefazolin 2 g iv PLUS metronidazole 500 mg iv	NR	NR		NR
Vaginal sling placement	NR	Cefazolin 2 g iv	Single dose of first-generation cephalosporin iv		Aminopenicillin and β-lactamase inhibitor 2 g iv (for prolapse)
Colporrhaphy	NR	Cefazolin 2 g iv	NR		NR
Cystoscopy	NR	Not recommended	NR		Not recommended
Urodynamic studies	NR	Not recommended	Not recommended ^5^		Not recommended
Issued	Surgical Antibiotic Prophylaxis Guideline at the Women’s in Parkville and in Sandringham 2020	Hellenic Society of Obstetricians and Gynecologists (HSOG) 2013	The guidelines on antibiotic application in gynecological Oncology (Serbia) 2005		Surgical Infection Prevention Guideline Writers Workgroup (SIPGWW) 2004
Hysterectomy: Laparotomy Laparoscopic Vaginal	Cefazolin 2 g iv within 60 min (ideally 15–30 min) before surgical incision (repeat dose if procedure > 4 h) PLUS Metronidazole 500 mg iv within 120 min (ideally 15–30 min) before surgical incision	Laparotomy: First- or second-generation cephalosporin OR Amoxicillin–clavulanic acid Laparoscopic and vaginal: First-generation cephalosporin (cefazolin)	Abdominal: 1 g of first generation cephalosporin (cephazolin, cefotetan, or cefoxitin) in one or maximum three doses. im 1 h or iv 20–30 min prior to the procedure ^13^ Vaginal: 2 g of cephalosporin with anaerobic activity (Cefotaxime or Cephazolin) OR amikacin 500 mg		Cefazolin, cefotetan, cefoxitin, or cefuroxime ^11^
Laparotomy without entry into bowel or vagina	Cefazolin 2 g iv within 60 min (ideally 15–30 min) before surgical incision (repeat dose if procedure > 4 h) PLUS Metronidazole 500 mg IV within 60 min (ideally 15–30 min) before surgical incision	NR	NR		NR
Laparoscopy without entry into bowel or vagina	Not recommended ^8^	Not recommended	Not recommended unless major and lengthy operation		NR
Hysteroscopy: Diagnostic Operative	Not recommended ^9^	Not recommended	NR		NR
Hysterosalpingography	Not recommended ^9^	Not recommended ^12^	NR		NR
Intrauterine device placement	Not recommended ^10^	Not recommended ^10^	NR		NR
Endometrial biopsy	Not recommended	Not recommended	NR		NR
Cervical tissue excision procedures (e.g., Large loop excision of transformation zone)	NR	NR	NR		NR
Surgical Termination of pregnancy	Screen patient for STIs: C. trachomatis, N. gonorrhoeae, M.genitalium, and bacterial vaginosis. Treat the woman and her partner(s) prior to termination of pregnancy.	Doxycycline 100 mg before and 200 mg after the procedure OR Doxycycline 400 mg before the procedure OR Metronidazole400 mg prior to the procedure and repeat 4 and 8 h after.	NR		NR
Urogynecological procedures (mid-urethral sling/TVT, colposuspension, and vaginal prolapse surgery +/− mesh/SSF)	Cefazolin 2 g iv within 60 min before surgical incision PLUS Metronidazole 500 mg IV within 120 min before surgical incision	First-generation cephalosporin	NR		NR
Urodynamic studies		Not recommended			
Issued	Spanish Society of Infectious Diseases and Clinical Microbiology (SEIMC) and Spanish Association of Surgeons (AEC) 2020	Enhanced Recovery After Surgery (ERAS) Society 2015, 2019, 2020, 2023	Italian Society of Gynecology and Obstetrics (SIGO) 2020	National Institute for Health and Care Excellence (NICE) 2019	Consensus Bundle on Prevention of Surgical Site Infections After Major Gynecologic Surgery 2017
Hysterectomy: Laparotomy Laparoscopy Vaginal	Cefazolin OR Cefotaxime OR amoxicillin– clavulanic acid	Cefazolin OR Amoxicillin– clavulanic acid			
Laparotomy without entry into bowel or vagina	Recommended for clean procedures with implantation of foreign material, clean–contaminated, or contaminated procedures	NR			
Laparoscopy without entry into bowel or vagina	Not recommended	Not recommended			
Hysteroscopy: Diagnostic Operative	NR	NR			
Hysterosalpingography	NR	NR			
Intrauterine device insertion	NR	NR			
Endometrial biopsy	NR	NR			
Cervical tissue excision procedures (e.g., Large loop excision of transformation zone)	NR	NR			
Uterine evacuation	Induced abortion or puerperal curettage: Doxycycline 100 mg po/2 h or iv prior to the procedure OR azithromycin 1 g po/iv plus metronidazole 500 mg po	NR			
Vulvectomy	NR	Single dose of antibiotic ^7^			
Urogynecology procedures	NR	NR			
Vaginal sling placement	NR	Insufficient evidence ^6^			
Colporrhaphy	NR	Insufficient evidence			
Cystoscopy	Not recommended (unless risk factors)	NR			
Urodynamic studies	Not recommended (unless risk factors)	NR			

^1^ Antibiotic therapy should be instituted if there is reason to suspect infection risk or if the findings upon the procedure indicate risk of infection, e.g., dilated fallopian tubes or pelvic inflammatory disease. ^2^ An alternative regimen is metronidazole 2 g po prior to the procedure PLUS 1 g azithromycin po prior to the procedure for patients at a high risk of infection. ^3^ Consider screening for STIs and treatment according to guidelines. If dilated tubes, prescribe appropriate antibiotics (e.g., doxycycline) ^4^ Healthcare professionals could consider screening for sexually transmitted infections in high-risk patients. ^5^ In patients at lower risk, with a background risk of UTI < 10% after urodynamics. ^6^ 93% of surgeons said they administered some form of antibiotic prophylaxis when graft material was used in prolapse surgery. ^7^ Despite the absence of randomized evidence supporting antimicrobial prophylaxis, given the high rate of SSI, antibiotic prophylaxis is recommended. Guidelines for vulvar and vaginal surgery: Enhanced Recovery After Surgery Society recommendations; DOI link: https://doi.org/10.1016/j.ajog.2020.07.039.(accessed on 28 August 2023) ^8^ If breach of bowel/uterine/vaginal cavity, same as laparotomy. ^9^ For patients with dilated tubes or a history of PID or tubal damage, administer azithromycin 1 g po. ^10^ Patients should be screened and treated for STIs prior to insertion: *C. trachomatis*, *N. gonorrhoeae*, *M. genitalium*, and bacterial vaginosis. ^11^ Metronidazole can be used as an alternative. Trovafloxacin, although still available in the United States, is recommended only for serious infections. ^12^ For patients with dilated tubes, administer doxycycline. ^13^ According to German Association of Gynecologists, it is recommended to administer 3 × 2 g of cephalosporin in radical hysterectomy with lymphadenectomy. Abbreviations: NR: not reported; iv: intravenously.

**Table 3:** Overview of antibiotic prophylaxis guidelines in special cases.

	The Royal Australian and New Zealand College of Obstetricians and Gynecologists (RANCOG)	American College of Obstetricians and Gynecologists (ACOG)	Society of Obstetricians and Gynecologists of Canada (SOGC)	Société Française d’ Anésthesie et de Réanimation (SFAR)
Non-severe hypersensitivity to penicillin	No difference	No difference	No difference	No distinction between non-severe and severe allergic reaction
Immediate or delayed severe hypersensitivity to penicillin (e.g., anaphylaxis or Stevens–Johnson syndrome)	Clindamycin 600 mg iv PLUS Gentamicin 2 mg/kg iv	Clindamycin 900 mg Metronidazole 500 mg PLUS ^1^ Gentamicin ^2^ 5 mg/kg Aztreonam 2 g	Clindamycin 600 mg iv erythromycin 500 mg iv metronidazole	Hysterectomy: Clindamycin 900 mg iv PLUS Gentamicin 5 mg/kg/day Prolapse surgery: Gentamicin 5 mg/kg/d PLUS Metronidazole 1 g
Obese patients	3 g iv cefazolin if patient >120 kg	3 g iv cefazolin if patient >120 kg	Consider doubling the dose in patients with morbid obesity (BMI > 35 kg/m^2^)	Double the dose in patients with BMI > 35 kg/m^2^
Prolonged procedure	Consider an additional dose if >3 h	Additional intraoperative doses administered at intervals of two times the half-life of the drug (after the first dose) Cefazolin should be redosed after 4 h	Additional dose may be administered 3 to 4 h after the initial dose if >3 h	Hysterectomy: Cefazolin if >2 h additional 1 g Cefamandole if >2 h additional 0.75 g Cefuroxime if >2 h additional 0.75 g Prolapse surgery: Aminopenicillin and β-lactamase inhibitor 1 g if >2 h In general, either additional dose or use of an antibiotic with longer half-life.
Excessive blood loss	No recommendation	A second dose may be appropriate when blood loss > 1500 mL	Additional dose may be administered 3 to 4 h after the initial dose if blood loss > 1500 mL	No recommendation
Colonization or high risk of colonization with MRSA	ADD Vancomycin 15 mg/kg iv	Hospital-recommended MRSA antibiotic prophylaxis protocol or addition of a single preoperative iv dose of vancomycin to the preoperative antibiotic prophylactic regimen		Patient screening debatable
	Spanish Society of Infectious Diseases and Clinical Microbiology (SEIMC) and Spanish Association of Surgeons (AEC)	Enhanced Recovery After Surgery (ERAS) Society	National Institute for Health and Care Excellence (NICE)	Surgical Antibiotic Prophylaxis Guideline at the Women’s in Parkville and in Sandringham
Non-severe hypersensitivity to penicillin	No distinction between non-severe and severe allergic reaction	No distinction between non-severe and severe allergic reaction		Use of cephalosporins can be considered
immediate or delayed severe hypersensitivity to penicillin (e.g., anaphylaxis or Stevens–Johnson syndrome)	Clindamycin PLUS gentamycin Vancomycin PLUS gentamycin	Standard surgical antibiotic prophylaxis including cefazolin or ertapenem ^4^		Avoid penicillin and cephalosporins Clindamycin 600 mg iv within 120 min before skin incision PLUS Gentamicin 2 mg/kg IV over 3–5 min within 120 min before skin incision
Obese patients	May require higher initial doses ^3^	Increase dose in patients with BMI > 35 kg/m^2^ or >100 kg		
Prolonged procedure	Additional dose if the surgical procedure exceeds 2 times the half-life of the antibiotic. Additional dose at 3 h for cefazolin or other antibiotic with similar half-life.	Additional dose after 1–2 times the half-life of the antibiotic in prolonged operations (e.g., 3 h for cefazolin; half-life 1.8 h)	Additional dose of antibiotic prophylaxis when the operation is longer than the half-life of the antibiotic administered	Additional dose if the surgical procedure exceeds 2 times the half-life of the antibiotic.
Excessive blood loss	Additional dose if blood loss > 1500 mL in adults or >25 mg/kg in children	Additional dose if blood loss > 1500 mL		
Colonization or high risk of colonization with MRSA	Consider use of glycopeptide			Cefazolin 2 g iv within 60 min before skin incision PLUS vancomycin 15 mg/kg iv, 15 to 120 min before skin incision.
	Italian Society of Gynecology and Obstetrics (SIGO)	Hellenic Society of Obstetricians and Gynecologists (HSOG)	Consensus Bundle on Prevention of Surgical Site Infections After Major Gynecologic Surgery	Surgical Infection Prevention Guideline Writers Workgroup (SIPGWW)
Non-severe hypersensitivity to penicillin			Consider patient-specific antibiotic regimen	
Immediate or delayed severe hypersensitivity to penicillin (e.g., anaphylaxis or Stevens–Johnson syndrome)		Clindamycin 600 mg iv PLUS erythromycin 500 mg iv	Consider patient-specific antibiotic regimen	Clindamycin PLUS gentamicin, aztreonam, or ciprofloxacin Metronidazole PLUS gentamicin or ciprofloxacin Clindamycin A 750 mg dose of levofloxacin can be substituted for ciprofloxacin
Obese patients		Double the dose if BMI > 35 kg/m^2^	Consider patient-specific antibiotic regimen	
Prolonged procedure	Additional dose if the surgical procedure exceeds 2 times the half-life of the antibiotic.	Additional dose after 3–4 h if the procedure lasts longer than 3 h	Additional dose if the procedure lasts longer than 2–3 h (redose at 1–2 times the half-life of the drug)	
Excessive blood loss	Additional dose if excessive blood loss (>1500 mL)	Additional dose after 3–4 h if blood loss > 1500 mL	Additional dose if excessive blood loss (>1500 mL)	
Colonization or high risk of colonization with MRSA			Consider patient-specific antibiotic regimen	Colonization: Vancomycin High risk: No evidence about the use of vancomycin

^1^ Ciprofloxacin 400 mg is an additional effective alternative if both gentamicin and aztreonam are not acceptable. ^2^ Dosage is based on the patient’s actual body weight. If the patient’s actual weight is more than 20% above ideal body weight (IBW), the “dosing weight” (DW) can be determined as follows: DW = IBW + 0.4 (actual weight-IBW). ^3^ Pharmacokinetic parameters may be higher in obese patients, but often not proportional to total body weight. ^4^ Nelson G, Fotopoulou C, Taylor J, Glaser G, Bakkum-Gamez J, Meyer LA, Stone R, Mena G, Elias KM, Altman AD, Bisch SP, Ramirez PT, Dowdy SC. Enhanced recovery after surgery (ERAS^®^) society guidelines for gynecologic oncology: Addressing implementation challenges—2023 update. Gynecol Oncol. June 2023;173:58–67. https://doi.org/10.1016/j.ygyno.2023.04.009. (accessed on 31 August 2023) Epub 21 April 2023. PMID: 37086524. Written in red: for obstetrics [15].

**Table 4:** Time of administration of prophylactic antibiotics: overview of guidelines.

The Royal Australian and New Zealand College of Obstetricians and Gynecologists (RANCOG)	At least 30 min prior to incision
American College of Obstetricians and Gynecologists (ACOG)	Within 1 h from skin incision (e.g., cefazolin). For quinolones or vancomycin, the infusion should begin before up to 2 h
Society of Obstetricians and Gynecologists of Canada (SOGC)	15–60 min prior to incision
Société Française d’ Anésthesie et de Réanimation (SFAR)	30 min prior to incision. For vancomycin, the infusion must be finished at least 30 min prior to the procedure.
Spanish Society of Infectious Diseases and Clinical Microbiology (SEIMC) and Spanish Association of Surgeons (AEC)	For βlactams (e.g., penicillin and cephalosporins such as cefazolin, cefoxitin, and cefuroxime) within 60 min from incision For vancomycin, aminoglycosides, or fluoroquinolones, intravenous infusion should begin 90 min prior to incision.
Enhanced Recovery After Surgery (ERAS) Society	Within 60 min from skin incision.
Hellenic Society of Obstetricians and Gynecologists (HSOG)	15–60 min prior to incision
National Institute for Health and Care Excellence (NICE)	Consider the timing and pharmacokinetics (for example, the serum half-life) and necessary infusion time of the antibiotic.
The guidelines on antibiotic application in gynecological Oncology (Serbia)	Intramuscularly 1 h or intravenously 20–30 min prior to the surgery.
Surgical Antibiotic Prophylaxis Guideline at the Women’s in Parkville and in Sandringham	Within 15–120 min from skin incision depending on the procedure and the antibiotic used.
Italian Society of Gynecology and Obstetrics (SIGO)	within 120 min from the incision, considering the half-life of the antibiotic
Consensus Bundle on Prevention of Surgical Site Infections After Major Gynecologic Surgery	Within 60 min before skin incision (most effective if administered 0–30 min before skin incision). Within 120 min for antibiotics that require slow infusion.
Surgical Infection Prevention Guideline Writers Workgroup (SIPGWW)	Within 60 min from incision. For fluoroquinolone or vancomycin, the infusion should begin within 120 min before incision
European Society of Gynecological Oncology (ESGO)	2 h time window before surgical incision, while considering the half-life of the antibiotic. Repeat intraoperative dosing of the antibiotic prophylaxis should be performed depending on the half-life time of the antibiotic and the duration of the surgery.

## Data Availability

No new data were created or analyzed in this study. Data sharing is not applicable to this article.
